# Statistical Inference of Selection and Divergence from a Time-Dependent Poisson Random Field Model

**DOI:** 10.1371/journal.pone.0034413

**Published:** 2012-04-03

**Authors:** Amei Amei, Stanley Sawyer

**Affiliations:** 1 Department of Mathematical Sciences, University of Nevada Las Vegas, Las Vegas, Nevada, United States of America; 2 Department of Mathematics, Washington University in St.Louis, St.Louis, Missouri, United States of America; Hungarian Academy of Sciences, Hungary

## Abstract

We apply a recently developed time-dependent Poisson random field model to aligned DNA sequences from two related biological species to estimate selection coefficients and divergence time. We use Markov chain Monte Carlo methods to estimate species divergence time and selection coefficients for each locus. The model assumes that the selective effects of non-synonymous mutations are normally distributed across genetic loci but constant within loci, and synonymous mutations are selectively neutral. In contrast with previous models, we do not assume that the individual species are at population equilibrium after divergence. Using a data set of 91 genes in two *Drosophila* species, *D. melanogaster* and *D. simulans*, we estimate the species divergence time 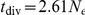 (or 1.68 million years, assuming the haploid effective population size 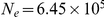 years) and a mean selection coefficient per generation 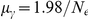. Although the average selection coefficient is positive, the magnitude of the selection is quite small. Results from numerical simulations are also presented as an accuracy check for the time-dependent model.

## Introduction

Mutation, selection, and genetic drift are important forces that shape pattern of genetic polymorphism within and between species [1]. McDonald and Kreitman first used a 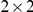 contingency table to test differences in selection between silent and amino acid replacement sites [2]. Data from the *Adh* gene encoding alcohol dehydrogenase in *Drosophila* suggested that adaptive fixation of selectively advantageous mutations was the cause of a statistically significant number of excess replacement substitutions. A quantitative theory for the amount of selection between two recently diverged species was developed by Sawyer and Hartl [3], who developed a sampling theory in which the set of frequencies of mutant sites is modeled as a Poisson random field (PRF). This theory was applied to the sample configurations of nucleotides in the *Adh* gene in *Drosophila* and led to maximum likelihood estimates of silent and replacement mutation rates, an average selection coefficient, and the species divergence time. Bayesian methods have proven useful for data sets with multiple genetic loci. Bustamante et al. [4] introduced a hierarchical Bayesian fixed effects model in which selective intensities of new replacement mutations are constant within genetic loci, but are normally distributed across genes. Application of this model using Markov chain Monte Carlo (MCMC) simulations yielded evidence for predominantly beneficial gene substitutions in *Drosophila* but detrimental substitutions in the mustard weed *Arabidopsis*. Sawyer et al. [5], [6] extended this model to a Bayesian random effects model in which selective effects of non-synonymous mutations are normally distributed within genes, while, as in Bustamante et al. [4], within-locus means are normally distributed across genetic loci. Abel [7] considered similar models with more heavy-tailed distributions within loci (specifically, Laplace and chi-square distributions) and found similar numerical results.

Although the PRF model of Sawyer and Hartl [3], [5], [6] provides robust estimates [7]–[12] for mutation and selection parameters, numerical simulations have shown that estimate of species divergence time is somewhat biased, particularly for small divergence time [7]. This is due to the model assumption that the two species are individually at mutation-selection-drift equilibrium after divergence. Recently, we have derived a “time-dependent” PRF model that removes this equilibrium assumption [13] (see the next section for details). Williamson et al. [12] proposed a time-inhomogeneous PRF model to make inference about constant selection and population growth simultaneously. They applied the model to site frequency spectrum of 301 human genes and showed a strong evidence for recent population growth. Later, Boyko et al. [9] extended the site-frequency spectrum based PRF approach to allow for simultaneous inference of demography and a distribution of fitness effects among newly arising mutations. The application of their method to a Single Nucleotide Polymorphism (SNP) data of 20 European Americans and 15 African Americans showed evidence of an ancient population expansion in the sample of African population and a relatively recent bottleneck in the sample of European American population. Given the estimates of demographic parameters, they made inference of the distribution of the selection effects. Both studies are based on maximum likelihood methods and only applied to a single population. In order to make inference about both selective effects and species divergence time, we developed a hierarchical Bayesian framework for sample configuration formulas derived from the time-dependent PRF model that contrasts the number of silent and replacement polymorphisms within species with that of fixed differences between species. We applied the model to 91 genes in African populations of *D. melanogaster* and *D. simulans* (Pröschel et al. [14]) and find that a large proportion of newly arising amino acid replacement mutations observed as polymorphisms are subject to weak positive selection. The model is first tested on a set of simulated data. Estimates of mutation and selection parameters are reasonably accurate. In particular, the point estimate of species divergence time matches the true parameter value almost perfectly for each simulated data. This shows the power of the time-dependent model in estimating species divergence time.

## Methods

A 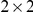 contingency table consisting of the number of fixed differences and polymorphisms at silent and replacement sites is called a MacDonald-Kreitman table and also a DPRS table. Assuming time equilibrium and independence among nucleotide sites, or equivalently linkage equilibrium, the four entries in a DPRS table can be regarded as independent Poisson random variables whose expected values can be derived from the fixation flux and limiting distribution of polymorphic nucleotide substitutions [3]. In the time-dependent case, we define two types of polymorphisms [3]. A site is a *legacy polymorphism* if the ancestors of the sequences in the DNA alignment were polymorphic at the time of divergence. The site is a *new polymorphism* if the polymorphism is caused by one or more mutations since the time of divergence. New polymorphisms can only show up in one species while legacy polymorphic sites can be polymorphic in one or both species. A natural extension of the DPRS table is a 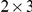 contingency table, called the DOHRS table, that has columns for two different types of polymorphisms. Specifically, we define 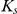 as the number of silent sites that are fixed differences between a pair of species in a sample (that is, monomorphic within samples but polymorphic between samples), 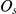 as the number of silent sites that are polymorphic in only one sample, and 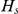 as the number of silent sites that are polymorphic in both samples [13]. We use 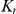, 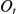, and 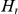 as the corresponding counts for amino acid replacement sites. Let 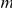 and 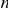 denote the number of aligned DNA sequences from the two species (which we assume to have the same haploid effective population size 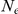) and let 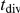 be the scaled divergence time since the time that the two populations diverged. For each locus, we use 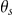 and 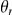 to represent, respectively, the scaled synonymous and non-synonymous mutation rates and 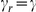 the scaled selection coefficient of a non-synonymous mutation. Synonymous mutations are assumed to be selectively neutral, i.e. 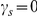, and unaffected by hitchhiking and other linkage-mediated effects. The parameters 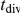, 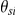, 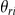, and 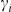 (where we now indicate the locus explicitly) are all scaled in terms of the haploid effective population size 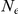 [13]. Assuming independence among sites, constant and equal effective population sizes 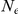 for both species, and no migration between species, the counts 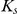, 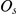, 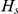, 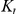, 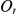, and 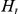 are independent Poisson random variables with means given, in a united form, by
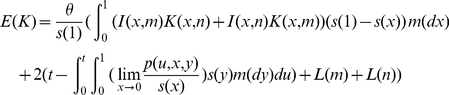
(1)

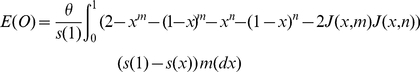
(2)

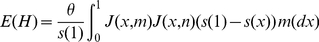
(3)where
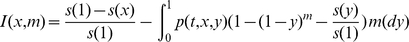


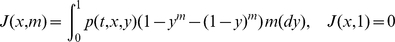


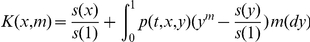


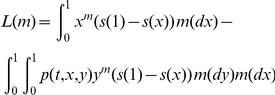
with 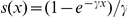, 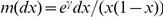 for replacement sites and 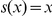, 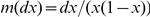 for silent sites.

The function 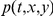 in these expressions is a smooth symmetric function of its arguments such that, for any continuous function 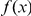 on 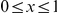, the integral 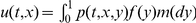 is the solution of the following diffusion equation
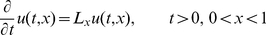
(4)

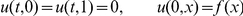
where 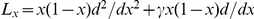 (see details in [13]).

At each locus, the theoretical expectations Eqs.(1)–(3) of the six Poisson counts (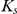, 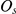, 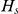, 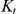, 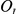, and 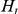) for a single DOHRS table depend on four parameters (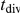, 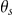, 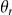, and 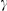), where 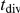 is a global parameter shared by all loci and the rest are locus specific parameters. The goal of this study is to use Bayesian methods to estimate genetic parameters based on a set of DOHRS tables of aligned gene sequences from a pair of closely related species. We assume that all non-synonymous mutant nucleotides at the 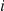
th locus have the same selection coefficient 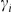. Across loci, the 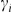 are normally distributed with mean 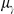 and variance 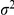. In our Bayesian framework (as in [4]), we assign an inverse-gamma-normal distribution as a joint prior distribution of the mean 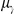 and variance 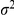, gamma distributions with given parameters as prior distributions of the two types of mutation rates 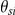 and 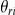, and a uniform distribution for the divergence time 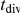. In standard Bayesian notation,
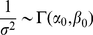
(5)

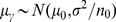
(6)

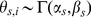
(7)

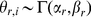
(8)

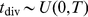
(9)All hyperparameters 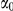, 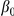, 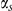, 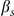, 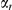, 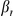, 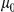, and 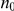 are chosen to be small (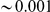) so as to be “uninformative” and 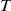 is a fixed large value. The full likelihood, based on the sampling formulas in Eqs.(1)–(3) and the prior distributions in Eqs.(5)–(9), is given explicitly by
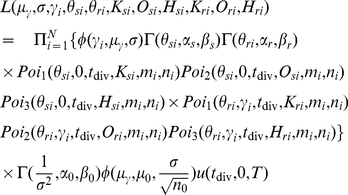
(10)where 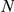 is the number of loci, 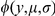, 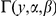, and 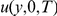 are respectively normal, gamma, and uniform densities, and
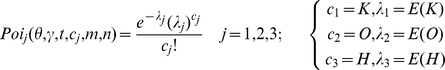
Integrals involving the transition density 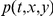 are estimated by Crank-Nicolson method ([12], [15]). Gauss-Legendre quadrature [15] is used for all other integrals. Finally, the posterior distributions of the genetic parameters given the Poisson counts in the DOHRS tables are obtained by means of Markov chain Monte Carlo simulations. In the implementation of MCMC simulations, convergence is assessed using traceplots as well as Gelman-Rubin statistics 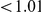 [16].

## Results

### Behavior on simulated data

We simulated 23 data sets each containing 30 genes as follows. For each data set, fixed values were assigned to the global parameters 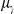, 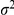, and 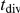. The locus specific parameters 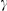, 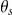, 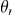, 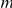, and 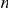 were generated from probability distributions. Specifically, at the 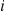
th locus, the selection coefficients 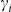 was sampled from the normal distribution with mean 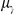 and variance 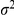, the two types of mutation rates 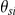 and 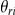 were drawn from two continuous uniform distributions with given ranges, and the number of DNA alignments 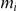 and 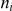 were taken from two discrete uniform distributions with specified ranges. For each locus, expected values for the Poisson counts 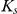, 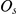, 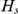, 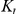, 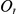, and 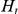 were calculated using Eqs.(1)–(3) with the given parameters. We then sampled six numbers from the Poisson distributions with calculated means to make up entries of each DOHRS table. Each simulated data set has 30 DOHRS tables.

**Figure 1:**
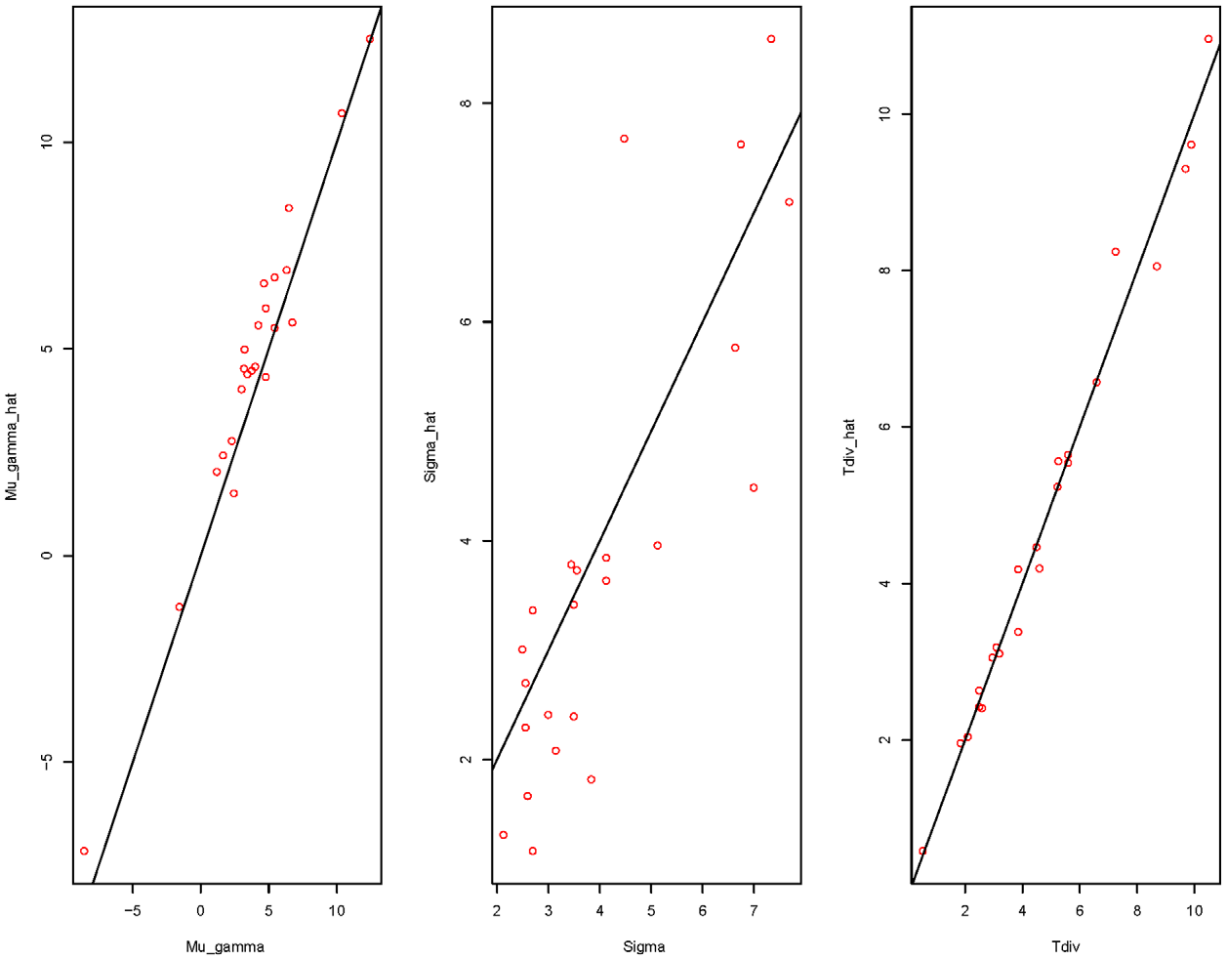
Comparisons of the estimated parameters with their corresponding true values based on 23 simulated data sets. Three plots are 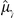 vs 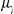, 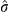 vs 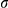, and 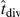 vs 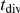 respectively. The selection coefficient 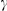 is assumed to be normally distributed with mean 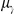 and variance 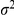 and 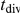 is the species divergence time.

**Figure 2:**
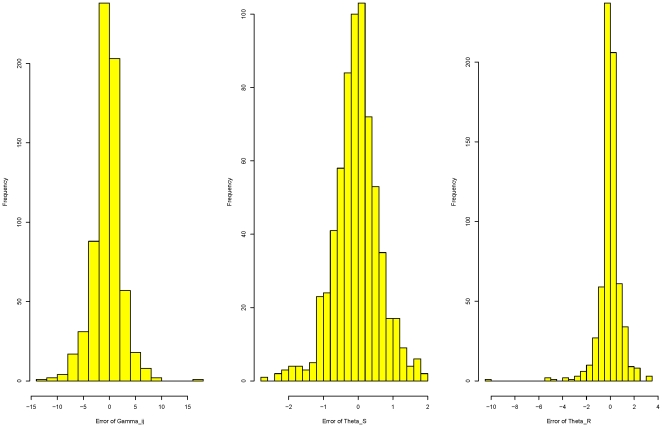
Histograms of estimation errors for selection coefficient ( 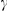
 **), silent mutation rate (** 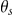 **), and replacement mutation rate (** 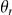 **) using 23 simulated data sets each containing 30 genes.**

As a check of accuracy of the time-dependent PRF model, estimated values of the parameters for each data set were compared with the given values. As shown in Figure 1, estimates of 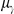 and 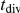 lie closely to their given values. The differences between estimates and true values for 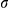 are small for small values of 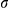 and increase as 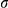 goes large. The 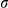 estimates may get improved by increasing the number of loci contained in each data set. Estimation errors of locus specific parameters are presented in Figure 2. These are histograms of 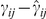, 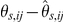, and 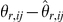 for 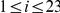 and 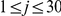 respectively. The results show that the point estimates for the two types of mutation rates are quite accurate. For the selection coefficients, the 95% posterior credible intervals obtained via MCMC algorithm cover the true parameters most of the time though the point estimates look less precise. Note that each 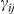 estimate is based on a single DOHRS table.

### Results on real data

**Figure 3:**
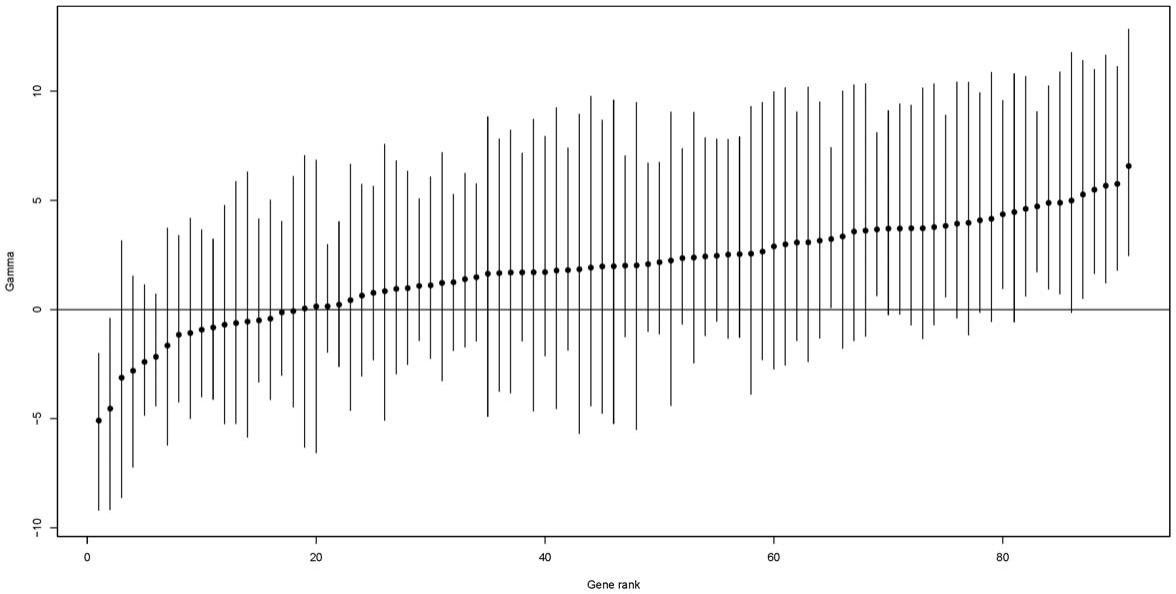
Estimated selection parameter ( 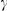
 **) for each gene with the loci sorted by the values of the estimates (medians).** Error bars represent 95% credible intervals.

We next applied our method to data of Pröschel et al. [14]. This consists of the coding sequences of 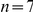 to 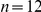 alleles of each of 91 autosomal genes in *Drosophila melanogaster* collected from a population near Lake Kariba, Zimbabwe. A single highly-inbred line of *D. simulans* (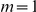) from Chapel Hill, North Carolina was used as a comparison of divergence [17]. After disregarding the first 20,000 burn-in iterations of MCMC simulations, estimates of parameters are obtained from 10,000 samples taken every 10 iterations. Scaled to the haploid population size, point estimates (median) and 95% credible intervals for the global parameters are 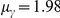 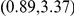, 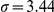 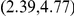, and 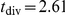 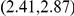. Selection coefficients for the 91 genes are estimated by medians of their posterior distributions. The medians and corresponding 95% credible intervals appear in Figure 3, with the loci sorted by the medians.

Among the 91 *Drosophila* genes, 73 have their median 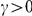 and 13 credible intervals are entirely positive (do not overlap 0). Although the mean amino acid replacement mutation that could contribute to polymorphism or divergence in *Drosophila* is beneficial, the magnitude of the selective intensity is small. Based on our estimates, 48% of the non-synonymous mutations have 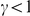, 84% have 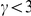, and 99% have 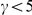. Assuming a haploid effective population size of 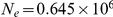 years [3], our estimate of 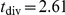 implies a species divergence time of 1.68 Myr (million years) between *D. melanogaster* and *D. simulans*. This value is almost in the middle of a range 0.8–3Myr [18], [19]. In contrast, the time-independent fixed effects model of [4] estimates 4.46 (median) with 95% credible interval (4.06, 5.00) for this data set and the time-independent random effects model of [5], [6] yields 4.47 and (4.06, 4.93).

**Figure 4:**
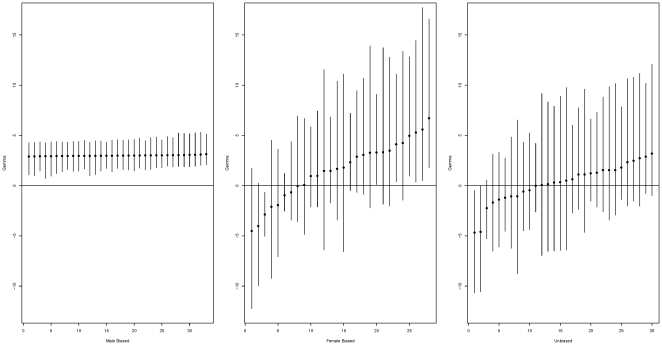
Estimated selection parameter ( 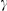
 **) for male-biased, female-biased, and sex-unbiased genes with the loci sorted by the values of the estimates (medians).** Error bars represent 95% credible intervals. Because selection coefficients are fitted jointly to a Gaussian distribution, uncertainties can be highly correlated. This is particularly visible for male-biased genes, where the uncertainty on the mean selection coefficient is larger than the estimated standard deviation.

Based on the difference of gene expression level between males and females (or between testes and ovaries), Pröschel et al. divided the data set into 33 male-biased, 28 female-biased, and 30 sex-unbiased genes [14]. We applied the time-dependent model to the three types of genes and means and standard deviations of the posterior distributions of the scaled selection coefficients are presented using their medians and 95% credible intervals. They are 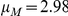 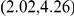, 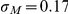 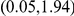, 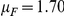 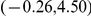, 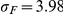 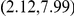, and 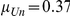 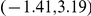, 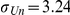 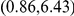 for male-biased, female-biased, and sex-unbiased genes respectively. Selection coefficients for individual genes of the three types are presented side by side, in Figure 4, using their median estimates and 95% credible intervals. According to our estimates, there is strong evidence that positive selection occur more often among sex-biased genes (both male and female) than among sex-unbiased genes. Specifically, the selection coefficients 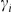 for male-biased genes, with an estimated normal distribution of mean 2.98 and standard deviation 0.17, show an almost uniform signal of adaptive selection. However, since we do not have information about linkage disequilibrium between these genes, we cannot exclude that this is simply a consequence of linkage between genes. In contrast, female-biased genes experience more variance in the direction of selection based on their estimated selection coefficients which vary from -4.52 to 6.70. On average, the selective effect for sex-unbiased genes are nearly neutral with a moderate size of variation (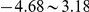). In their original paper, Pröschel et al. estimated average strength of selection for non-synonymous mutations within each group of genes using a time-independent fixed effects PRF model [14]. After excluding all low-frequency (singleton) polymorphisms, the mean selection parameter 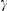 were estimated to be 2.0 and 1.8 for male- and female- biased genes respectively, while the mean 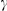 for sex-unbiased genes was −0.1. These results are quite consistent with our estimates. Later, Baines et al. studied effects of X-linkage on sex-biased gene evolution using a time-independent random effects PRF model [20]. They analyzed DNA sequence polymorphism and divergence in 45 X-linked genes for which 17 are male-biased, 13 are female-biased, and 15 are sex-unbiased genes and found evidence for adaptive evolution in both group of sex-biased genes. The estimated mean selection coefficients for male-biased, female-biased, and sex-unbiased genes are respectively 4.7, 2.5, and −0.8 using all polymorphic sites and 7.4, 2.1, and 0.5 after removal of singleton polymorphisms.

## Discussion

The classical Bayesian model [4]–[6], fixed effect or random effects, assumes that the two daughter populations are immediately at mutation-selection-drift equilibrium after species divergence. Knowing that this assumption may be biologically unrealistic, we apply a previously developed time-dependent Poisson random field model to DNA sequences data to make inferences about selection, mutation, and species divergence. The results of this study suggest that a majority of newly-arisen non-synonymous mutations observed as polymorphisms is beneficial, although the magnitude of selection is very small, which is consistent with the conclusion drawn by Bustamante et al. [4] where a time-independent fixed effects PRF model was applied to 34 genes from *D. melanogaster* and *D. simulans*. Based on the results of Markov chain Monte Carlo simulations, they estimated the selection coefficient 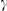 for each individual gene and concluded that “the average amino-acid replacement that is polymorphic or fixed in *Drosophila* is beneficial”. The set of 91 *D. melanogaster* genes studied here has previously been analyzed in a time equilibrium random effects PRF model [6]. Specifically, they assumed that the selective effect of each non-synonymous mutation, 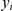, is normally distributed with mean 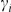 and variance 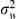 but the mean selection coefficient 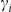 within a gene varies from one gene to the next, according to a normal distribution with mean 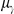 and variance 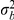. Scaled to the diploid population size, they estimated the mean selection coefficient 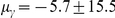, within- and between-locus standard deviations 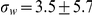 and 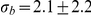 respectively. Their analysis suggested that 95% of all replacement mutations that could contribute to polymorphism or divergence are deleterious. On the other hand, majority of fixed differences between species are positively selected. The difference between our estimate of the mean selective effect of newly arisen non-synonymous mutations and that of Sawyer et al [6] is due to the assumption imposed on the distribution of the selective effects within a locus. It is biologically more realistic to model selective effect within a gene as random variable, as in [6], instead of constant. However assuming mutation-selection-drift equilibrium is artificial and it may bias the estimates of selective effects. In contrast, we put the species divergence time explicitly into the model to also make it biologically more reasonable. The Bayesian framework that we have applied in this study assumes that the selection intensity 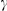 is same for each coding sequence but distributed normally with a fixed mean and variance across loci. This assumption is somewhat artificial but it is still meaningful for the original purpose of inferring polymorphism and divergence based on the newly proposed time-dependent PRF model. To conquer the disadvantages of the two models as well as to be able to estimate the fraction of amino acid fixations that are driven by positive selection, we are developing a more sophisticated time-dependent random effects model and its application to simulated data as well as to real data will appear in a future publication. Because the time of divergence is explicitly built into the model, we can estimate the value of the divergence time precisely and hence, it will help us to distinguish between fixations of beneficial mutations in a short period of time and fixations of deleterious mutations over a long period of time. The PRF model was derived under the assumption of independence among nucleotide sites. Due to the fact that high levels of recombination between nucleotides results in nearly independent assortment, whereas tight linkage is caused by low rates of recombination, it is equivalent to assume that nucleotide sites are at linkage equilibrium. For estimates of the mean selection coefficients, simulations have shown that methods based on Poisson random field for multi-locus data are relatively robust to the violation of this assumption([7], [9], [21], [22]). The effect of linkage on the overall shape, in particular, the variance, of the distribution of the selective coefficients needs to be analyzed as part of the model validation. The model also assumes that individual species have constant and equal population sizes. However changes in varying recombination rates and demographic history of the population such as population expansion and bottlenecks may result in changes of population size that could affect the parameter estimates and hence confound the interpretation of polymorphism and divergence ([2], [23]–[26]). The use of African *Drosophila* sample can avoid some of the demographic complexity ([27], [28]). As we mentioned earlier, one highly-inbreed line from *D. simulans* was used as a comparison of divergence. Although the high inbreeding ratio contradicts with the model assumption of equal population sizes, the use of a single line from second species minimizes the effect caused by this contradiction. Further study need to be conducted to check the robustness of the model to deviations from the assumptions.
